# CCR7 Regulates Cell Migration and Invasion through JAK2/STAT3 in Metastatic Squamous Cell Carcinoma of the Head and Neck

**DOI:** 10.1155/2014/415375

**Published:** 2014-10-27

**Authors:** Fa-Yu Liu, Jawad Safdar, Zhen-Ning Li, Qi-Gen Fang, Xu Zhang, Zhong-Fei Xu, Chang-Fu Sun

**Affiliations:** ^1^Department of Oromaxillofacial-Head & Neck Surgery and Department of Oral and Maxillofacial Surgery, School of Stomatology, China Medical University, No. 117 Nanjing Bei Jie, Heping District, Shenyang, Liaoning 110002, China; ^2^Department of Head and Neck, Henan Tumor Hospital, Zhengzhou University, 127 Dongming Road, Zhengzhou, Henan 450008, China

## Abstract

Squamous cell carcinoma of the head and neck (SCCHN) frequently involves metastasis at diagnosis. Our previous research has demonstrated that CCR7 plays a key role in regulating SCCHN metastasis, and this process involves several molecules, such as PI3K/cdc42, pyk2, and Src. In this study, the goals are to identify whether JAK2/STAT3 also participates in CCR7's signal network, its relationship with other signal pathways, and its role in SCCHN cell invasion and migration. The results showed that stimulation of CCL19 could induce JAK2/STAT3 phosphorylation, which can be blocked by Src and pyk2 inhibitors. After activation, STAT3 was able to promote low expression of E-cadherin and had no effect on vimentin. This JAk2/STAT3 pathway not only mediated CCR7-induced cell migration but also mediated invasion speed. The immunohistochemistry results also showed that the phosphorylation of STAT3 was correlated with CCR7 expression in SCCHN, and CCR7 and STAT3 phosphorylation were all associated with lymph node metastasis. In conclusion, JAk2/STAT3 plays a key role in CCR7 regulating SCCHN metastasis.

## 1. Introduction

The 5-year survival rate for patients with squamous cell carcinoma of the head and neck (SCCHN) is only 30%, mainly due to the frequent presence of metastasis at diagnosis [1]. The mechanisms leading to SCCHN metastasis are incompletely understood.

The CC chemokine CCL19 and its receptor CCR7, which regulate chemotaxis and the transendothelial migration of leukocytes during immune and inflammatory reactions, were recently observed to play an important role in the metastasis of various types of cancer [2–5]. We have also reported that CCR7 regulates cell migration and adhesion in metastatic squamous cell carcinoma of the head and neck by activating integrin, PI3K/cdc42, pyk2, and Src [6–15]. However, the signaling pathways controlling directional cell migration are not linear; rather, they integrate signals from a plethora of upstream switches into a molecular matrix, resulting in complex cellular responses. There may be other molecules in CCR7's signal pathway.

The JAK2/STAT3 pathway is critical for cytokine and growth factor-mediated responses regulating EMT biology in fibrogenesis and cancer [16]. The pathway widely participates in tumor metastasis and survival in various cancers, including colorectal cancer, breast cancer, and skin cancer [17–19]. The research in recent years has demonstrated that chemokines can also induce JAK2/STAT3 pathway activation. In small cell lung cancer, CXCL12 can stimulate JAK2/STAT3 constitutive phosphorylation, which is important in tumor cell growth and spreading [20]. In addition, in bladder cancer, CXCR7 can also activate the STAT3 pathway [21].

The goals of this study were to determine whether the JAK2/STAT3 pathway is activated by CCR7, the relationship with the other signal pathways activated by CCR7, and the role and the molecular mechanisms of the JAK2/STAT3 pathway in CCR7-regulated SCCHN metastasis.

## 2. Materials and Methods

### 2.1. Human Tumor Samples and Cell Lines

SCCHN tissue specimens were obtained from 78 patients via biopsy prior to chemotherapy or radiotherapy at the Department of Oral and Maxillofacial Surgery, School and Hospital of Stomatology, China Medical University. Ten samples of normal tissues adjacent to the benign tumor were chosen as controls. All clinical investigations were conducted according to the principles expressed in the Declaration of Helsinki. The study protocol was granted approval from the Ethics Committee of the China Medical University, and informed consent was obtained from the patients before surgery. PCI-4B and PCI-37B, which are well-characterized SCCHN cell lines that are derived from the metastatic lymph node of SCCHN patients, were kindly donated by the University of Pittsburgh Cancer Institute [22, 23]. The cells were cultured in DMEM medium (Invitrogen, Carlsbad, CA, USA) containing 10% fetal bovine serum (Gibco, Carlsbad, CA, USA), 100 U/mL penicillin G, and 100 U/mL streptomycin.

### 2.2. Reagents and Antibodies

CCL19 and CCR7 specific monoclonal antibody (mouse antihuman CCR7 antibody) were purchased from R&D System (Minneapolis, MN, USA), PP2 (Src inhibitor) was purchase from Santa Cruz Biotechnology (Dallas, TX, USA), LY294002 (PI3K inhibitor), Tyrphostin A9 (pyk2 inhibitor), and AG490 (JAK2 inhibitor) were purchased from Sigma (St. Louis, MO, USA). The anti-phospho-JAK2, anti-JAK2, anti-phospho-STAT3, anti-STAT3, antivimentin, and anti-E-cadherin were purchases from cell signaling technology (Danvers, MA, USA).

### 2.3. Immunohistochemical Staining and Evaluation

Sections were deparaffinized in xylene for 10 min, rehydrated through graded alcohols, immersed in 100% methanol containing 0.3% hydrogen peroxide for 40 min, placed in a microwave oven in a jar filled with 10 mM sodium citrate buffer (pH 6.0) for 10 min, and cooled at room temperature. Then sections were incubated with normal goat serum for 20 min, incubated with the primary antibody for 1 h, incubated with the linking reagent (biotinylated anti-immunoglobulin, Zymed, South San Francisco, CA, USA) at room temperature for 1 h, incubated with a complex of avidin DH and biotinylated enzyme (Zymed) for 30 min, and incubated with a medium consisting of an equal volume of 0.02% hydrogen peroxide and diaminobenzidine tetrahydrochloride (Zhongshan Ltd., Beijing, China) for 1 min in the dark. After chromogen development, sections were washed in water and counterstained with hematoxylin. The stained slides were investigated independently by two pathologists who had no knowledge of the clinical parameters and outcomes. All of these cells were scored as negative (−) (<10% or no staining), weak positive (+) (11–50%), positive (++) (51–75%), or strongly positive (+++) (>75%).

### 2.4. Western Blotting Analysis

Cells lysates were sonicated for 3 sec and centrifuged at 4°C and 14,000 rpm for 30 min. The supernatant was collected for protein quantification using the Bio-Rad Protein Assay dye reagent (Bio-Rad Laboratories, Richmond, CA, USA). Fifty micrograms of protein was size-fractionated through a 10% SDS-PAGE gel and transferred onto nitrocellulose filters. The filters were blocked (1% nonfat dry milk, 0.1% Triton X-100, 150 mM NaCl, 50 mM Tris [tris(hydroxymethyl)aminomethane] (pH 7.5)) and incubated with the primary antibody, which was diluted to a ratio of 1 : 1000. Nitrocellulose filters were incubated with horseradish peroxidase-conjugated secondary antibodies. Bands were visualized using the enhanced chemiluminescence system (Amersham Pharmacia Biotech, Piscataway, NJ, USA) and quantified by scanning densitometry using FlourChem V2.0 software.

### 2.5. Migration Assay

Disposable 24-well transwell inserts with an 8 *μ*m pore size were run in triplicate in DMEM with 0.5% (w/v) BSA. Aliquots of the chemokine CCL19 were added to the lower chamber at a concentration of 500 ng/mL. The inhibitor-pretreated PCI-4B and PCI-37B cell suspensions (2 × 10^5^) were placed in the top of the inserts. After 24 h of incubation, the cells on the upper surface of the inserts were removed with a cell harvester, and the membrane was washed with medium. Cells that penetrated the membrane were fixed with ice-cold methanol, stained with 0.5% crystal violet, photographed, and counted under the microscope. The mean ± standard deviations (SD) were recorded for each condition, and the migration index was calculated based on the control involving random migration.

### 2.6. Matrigel Invasion Assay

Cell invasion was quantified in vitro using Matrigel-coated semipermeable, modified inserts with a pore size of 8 *μ*m. The analysis of the Matrigel invasion assay was performed as described in the migration assay incubated with CCL19 for 36 h. The mean ± standard deviations (SD) were recorded for each condition, and the invasion index was calculated based on the control involving random invasion.

### 2.7. Scrape Wound-Healing Assay

SCCHN cells were plated in a 24-well plate at an initial density of 1.5 × 10^5^ cells/cm^2^. A uniform monolayer formed in 2-3 days. All wound-healing assays were performed in a serum-free medium. A micropipette tip was used to create a wound in the monolayer by scraping. The relative cell free area was calculated based on the control group.

### 2.8. Statistical Analysis

Data were expressed as the mean ± standard deviation (SD) of repeated assays. The correlation was analyzed using Spearman's test and *χ* ^2^ test. Significant differences between the two groups were evaluated using an unpaired Student's *t*-test. *P* values <0.05 were considered to be significant. All statistical analyses were performed with the software SPSS 11.0.

## 3. Results

### 3.1. CCR7 Stimulates the Phosphorylation of JAK2

**Figure 1:**
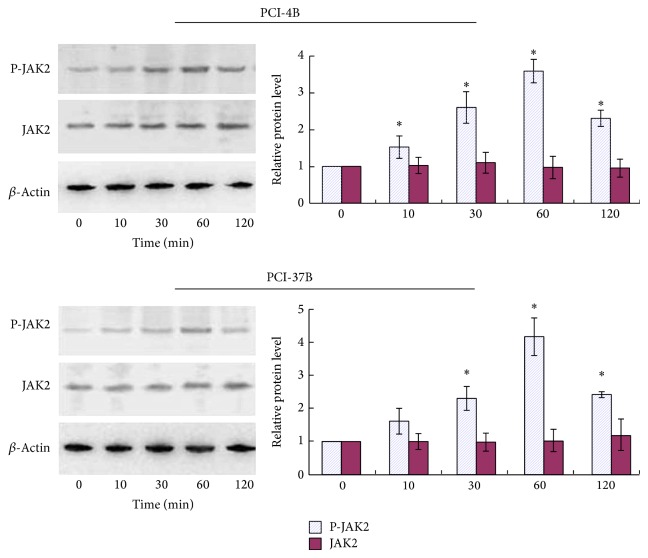
Western blotting analysis of JAK2 expression induced by CCL19 at different times. PCI-4B and PCI-37B cells were treated by CCL19 (200 ng/mL) for 0–120 min, and JAK2 total protein expression and phosphorylation protein expression were detected. The results are representative of three independent experiments. ^*^ *P* < 0.05 compared with the control group.

PCI-4B and PCI-37B cells were stimulated with CCL19 for various time periods and then lysed, and the lysates were analyzed by western blotting using Abs specific for the phosphorylated/active forms and total protein of JAK2. The results showed that stimulation with CCL19 resulted in phosphorylation of JAK2, but there was no contribution to the total JAK2 protein. The phosphorylation of JAK2 is time dependent, and it reached a maximum in 60 min, which is approximately 3-4-fold of baseline (Figure 1).

### 3.2. CCR7 Stimulates the Phosphorylation of STAT3

**Figure 2:**
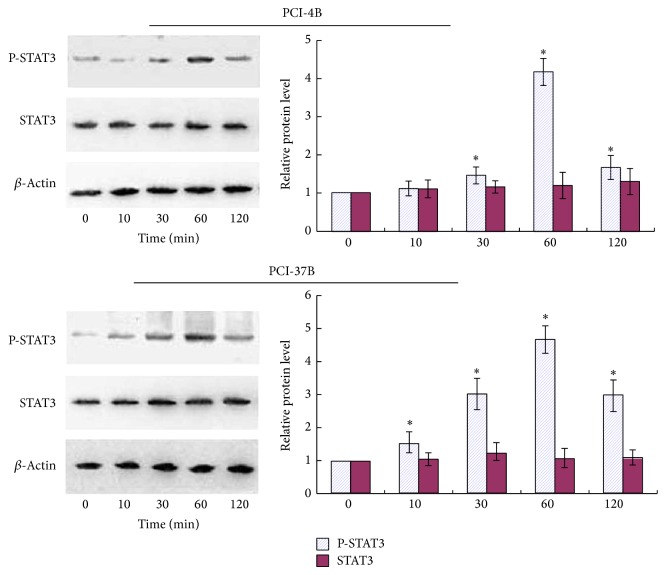
Western blotting analysis of STAT3 expression induced by CCL19 at different times. PCI-4B and PCI-37B cells were treated by CCL19 (200 ng/mL) for 0–120 min, and STAT3 total protein expression and phosphorylation protein expression were detected. The results are representative of three independent experiments. ^*^ *P* < 0.05 compared with control group.

As the primary downstream molecule of JAK2, STAT3 was also examined. It can be seen in Figure 2 that stimulation with CCL19 also resulted in phosphorylation of STAT3 and no contribution to the total STAT3 protein. The phosphorylation that occurred in 60 min is approximately 4-5-fold that of the control group.

**Figure 3:**
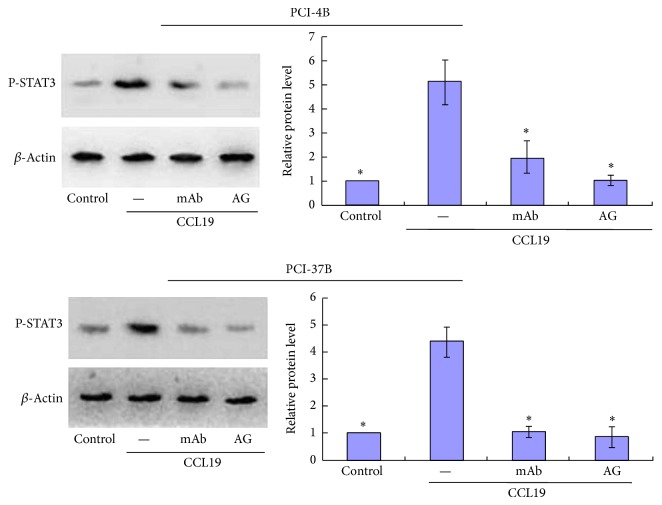
Western blotting analysis of the inhibitors' role in CCL19-induced STAT3 phosphorylation. PCI-4B and PCI-37B cells were pretreated by CCR7 mAb (10 *μ*g/mL) for 4 h or by AG490 (30 *μ*M) for 24 h and then by CCL19 (200 ng/mL, 60 min). Phosphorylation of STAT3 was detected by western blotting. The results are representative of three independent experiments. ^*^ *P* < 0.05 compared with the CCL19 group.

To further determine whether CCR7 regulates the activation of STAT3, PCI-4B and PCI-37B cells were pretreated with CCR7 mAb, an antibody that can neutralize the bioactivity of CCR7, and AG490, the JAK2/STAT3 inhibitor. Then, the cells were stimulated by CCL19, and activation of p-STAT3 was analyzed. The results showed that treatment with CCR7 mAb completely abrogated the CCL19-dependent activation of STAT3 (Figure 3), indicating that CCR7's activation stimulated the phosphorylation of STAT3 in SCCHN cells. As the inhibitor, AG490 can also block the activation of STAT3.

**Figure 4:**
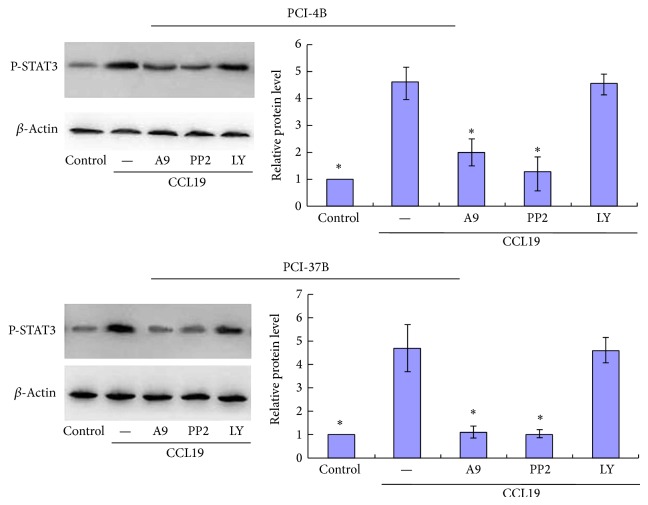
Western blotting analysis of Pyk2, Src, and PI3K inhibitors' roles in CCL19-induced STAT3 phosphorylation. PCI-4B and PCI-37B cells were pretreated by Tyrphostin A9 (5 *μ*M, 1 h), PP2 (20 *μ*M, 4 h), LY294002 (50 *μ*M, 4 h), and then CCL19 (200 ng/mL, 60 min). The phosphorylation of STAT3 was detected by western blotting. The results are representative of three independent experiments. ^*^ *P* < 0.05 compared with the CCL19 group.

Our previous results have shown that CCR7 can activate several signal pathways, including PI3K, Src, and pyk2 [7, 10, 15]. To analyze whether they are upstream pathways of JAK2/STAT3 in SCCHN, PCI-4B and PCI-37B cells were pretreated with PI3K inhibitor (LY294002), Src inhibitor (PP2), and pyk2 inhibitor (A9). The results showed that PP2 and A9 could blunt the increase of the phosphorylation of STAT3 induced by CCL19, with significant differences with the CCL19 group. In addition, the LY294002 played no role in STAT3 activation, without significant differences with the CCL19 group. This means that Src and pyk2 may be the upstream signal molecules of STAT3 (Figure 4).

### 3.3. JAK2/STAT3 Regulate CCR7-Dependent Migration and Invasion

**Figure 5:**
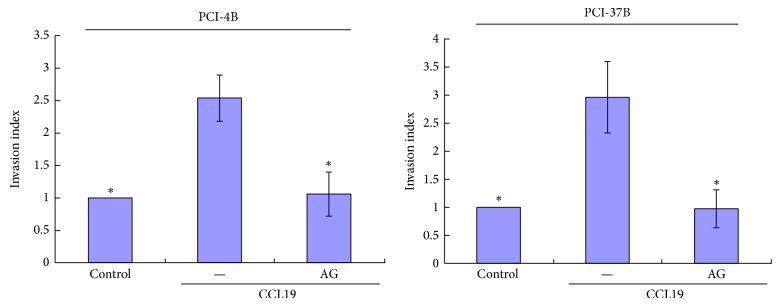
The role of JAK2/STAT3 inhibitor in CCL19-induced cell invasion. PCI-4B and PCI-37B cells were pretreated by AG490 (30 *μ*M) for 24 h and then by CCL19 (500 ng/mL, 36 h). The results are representative of three independent experiments. ^*^ *P* < 0.05 compared with the CCL19 group.

**Figure 6:**
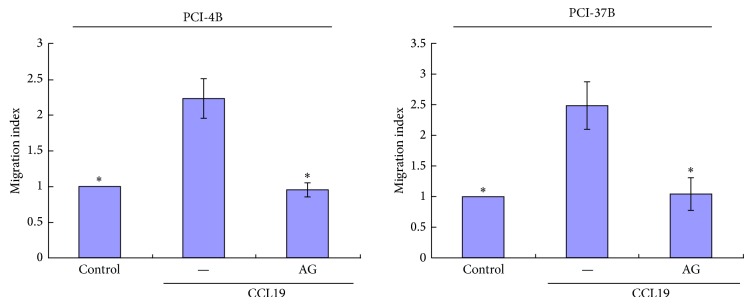
The role of JAK2/STAT3 inhibitor in CCL19-induced cell migration. PCI-4B and PCI-37B cells were pretreated by AG490 (30 *μ*M) for 24 h and then by CCL19 (500 ng/mL, 24 h). The results are representative of three independent experiments. ^*^ *P* < 0.05 compared with the CCL19 group.

Our previous results have showed that CCL19 induces PCI-4B and PCI-37B cell migration and invasion, and this role can be blocked by CCR7 mAb [7, 9]. In this study, we examined whether STAT3 is involved in regulating the migratory and invasive speed induced by CCR7 activation. The results showed that PCI-4B and PCI-37B cells exhibited a high invasion potential induced by CCL19, and this invasion potential was blocked by AG490 pretreatment and decreased almost to the baseline (Figure 5). The migration index was the same as the invasion index. That is, the CCL19-induced migration was significantly blocked by JAK2/STAT3 inhibitor (Figure 6).

**Figure 7:**
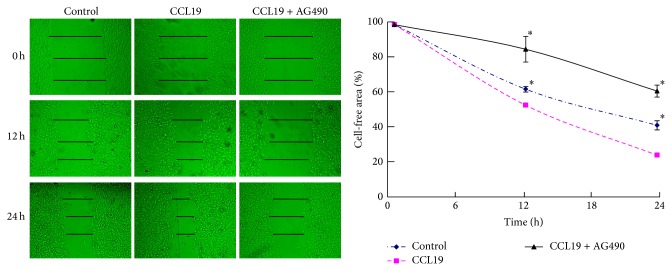
The role of JAK2/STAT3 inhibitor in CCL19-induced scrape wound-healing. PCI-37B cells were pretreated by AG490 (30 *μ*M) for 24 h and then by CCL19 (500 ng/mL), and the cell free area width was detected in 0–24 h. The results are representative of three independent experiments. ^*^ *P* < 0.05 compared with the CCL19 group.

We also used the scrape wound-healing assay, which requires both migration and proliferation of cells. The defined lesions were generated in subconfluent layers of cells, and the repopulation of denuded areas was studied. After 12 h, although the wounds in the control group and in the CCL19 group both started to close, the CCL19 group was significant. In addition, this difference was more significant at 24 h. When the inhibitors of JAK2/STAT3 were pretreated, the CCL19-induced cell migration and proliferation decreased significantly, and the free area was even larger than the control group (Figure 7). These results imply that the JAK2/STAT3 signal pathway may regulate CCR7-dependent migration and invasion.

### 3.4. JAK2/STAT3 Mediated the Expression Levels of E-Cadherin Induced by CCR7

**Figure 8:**
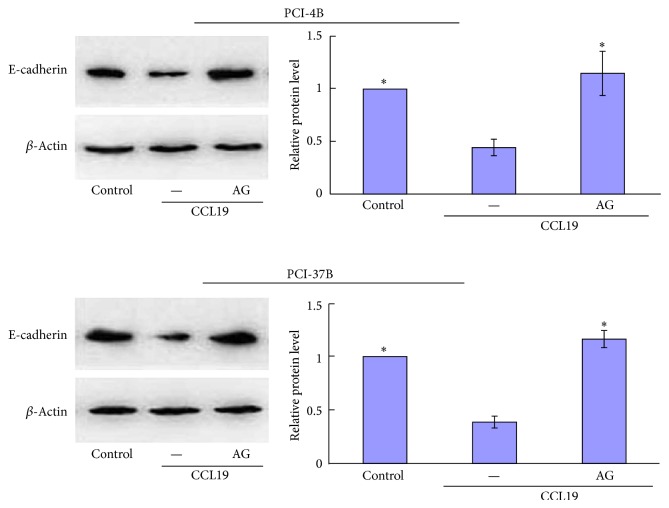
Western blotting analysis of the role of JAK2/STAT3 inhibitor in CCL19-induced E-cadherin. PCI-4B and PCI-37B cells were pretreated by AG490 (30 *μ*M) for 24 h and then by CCL19 (200 ng/mL, 30 min). E-cadherin expression was detected by western blotting. The results are representative of three independent experiments. ^*^ *P* < 0.05 compared with the CCL19 group.

**Figure 9:**
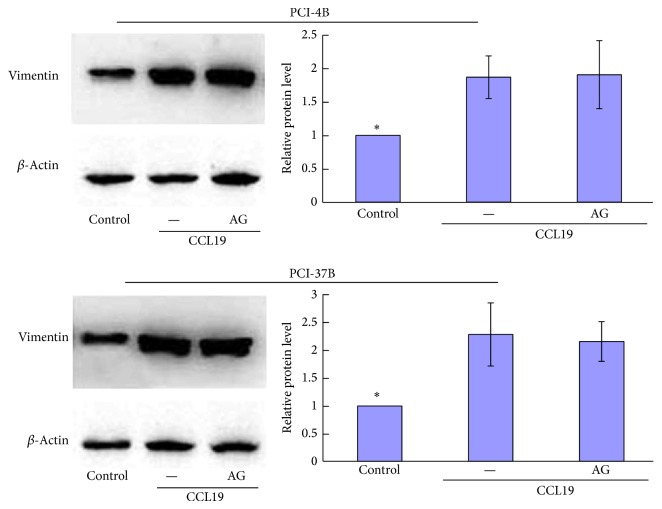
Western blotting analysis of the role of JAK2/STAT3 inhibitor in CCL19-induced vimentin. PCI-4B and PCI-37B cells were pretreated by AG490 (30 *μ*M) for 24 h and then by CCL19 (200 ng/mL, 30 min). Vimentin expression was detected by western blotting. The results are representative of three independent experiments. ^*^ *P* < 0.05 compared with the CCL19 group.

E-cadherin and Vimentin are known to be associated with the epithelial mesenchymal transition (EMT), which is a key point in tumor progression and generally participate various cells migration and invasion. Our previous results have showed that CCL19-treated PCI-4B and PCI-37B cells led to a significant increase in the level of vimentin protein and a significant decrease in the level of E-cadherin (the article is under review). In this study, we pretreated cells with AG490 and found that when the JAK2/STAT3 signal pathway was blocked, CCL19-induced low E-cadherin expression was increased significantly, even more than the control group, whereas no influence was observed for CCL19-induced vimentin expression (Figures 8 and 9). The results demonstrate that the CCR7-induced JAK2/STAT3 signal pathway regulates SCCHN cell migration and invasion via E-cadherin, without vimentin participating.

### 3.5. Phosphorylation of STAT3 Expressed by Immunohistochemical Staining Had a Significant Positive Correlation in Tumor Metastasis

**Figure 10:**
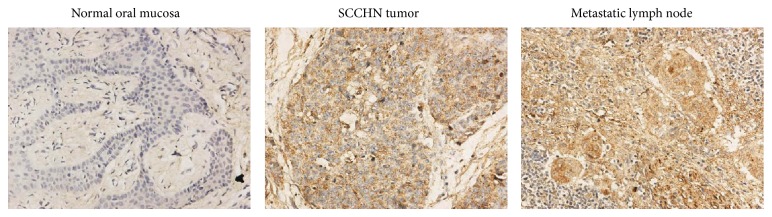
Immunohistochemical staining of STAT3 phosphorylation in normal tissue, an SCCHN primary tumor, and a metastatic lymph node.

In our previous study, CCR7 was examined in 78 specimens by immunohistochemical staining, and it was demonstrated that CCR7 is highly expressed in SCCHN tissue and is correlated with tumor metastasis [13]. In this study, we examined the phosphorylation expression of STAT3 in the same specimens. As Figure 10 shows, in normal tissues, p-STAT3 was not stained, whereas in primary SCCHN tissues, it was stained positively in nuclei, cytoplasm, or both, and in metastatic lymph nodes, the staining was even greater compared with the primary tumor. Of the 78 patients, 46 cases were positive for phosphorylation of STAT3 (46/78), and of the ten control cases, no cases were positive for phosphorylation of STAT3 (0/10). SCCHN and normal tissues exhibited a significant difference in the expression of phosphorylation of STAT3 (*P* < 0.05).  Table 1 summarizes the relationship between the phosphorylation of STAT3 and the clinicopathological factors of the 78 SCCHN patients. The expression of the phosphorylation of STAT3 was significantly correlated with cervical lymph node metastasis and clinical stage (*P* < 0.05). We also analyzed the relationship between CCR7 and p-STAT3 expression in these 78 specimens. The results showed a positive correlation between them (Table 2).

## 4. Discussion

STAT3, a molecular hub for diverse signaling pathways, such as cell cycle progression, apoptosis, angiogenesis, and immune evasion, has been identified as an oncogene and is constitutively activated in a variety of human malignancies [24]. The JAK2/STAT3 pathway is critical for cytokine and growth factor-mediated responses regulating EMT biology in fibrogenesis and cancer [16]. In primary breast cancer, interleukin-6 (IL-6) induced activation of its downstream effectors, JAK2 and STAT3, and this signaling played a critical and pharmacologically targetable role in orchestrating the composition of the tumor microenvironment that promotes growth, invasion, and metastasis [25]. Recently, an article about JAK2/STAT3 and chemokine receptors was published. In prostate cancer cells, the interaction of CCL2 and CCR2 induced STAT3 activation. Interruption of this CCL2/CCR2-STAT3 axis suppressed EMT and cell migration, resulting in better suppression of tumor growth and metastasis in a xenograft prostate cancer mouse model [26]. In our study, the C-C chemokine CCL19 was used to activate its receptor CCR7, and the JAK2/STAT3 was also phosphorylated, which can be blocked by CCR7 mAb, suggesting that JAK2/STAT3 is a downstream signal pathway of CCR7 in SCCHN. When this signal pathway is blocked by STAT3 inhibitor, the CCL19-induced cells' migration and invasion were also inhibited, demonstrating the key role of this pathway in SCCHN metastasis, as well as in other tumors.

CCR7 can activate several signal pathways in SCCHN, including PI3K, Src, and pyk2 [7, 10, 15]. PI3K, Src, pyk2, and STAT3 signal pathways are complex and interact with one another in different tumors. A research study reported that the inhibition of the activation of upstream JAK1, JAK2, and c-Src kinases suppressed the downstream STAT3 in human multiple myeloma cells [27]. Pyk2 is a member of the focal adhesion kinase family and can be activated by c-Src. In HeLa cells, along with c-Src, Pyk2 can facilitate EGFR-mediated STAT3 activation [28]. As two distinct regulatory networks, the PI3K pathway and STAT3 signaling have a functional link in some tumors [29]. In a glioblastoma cell line, the ectopic expression of PTEN, the regulator of PI3K/Akt, disrupted STAT3 signaling and resulted in growth retardation and senescence, whereas the LY294002, another inhibitor of PI3K/Akt, only induced transient dephosphorylation of STAT3 and rapidly restored it to its original level. Therefore, the authors believe that the STAT3 signal is not a downstream target of the PI3K/Akt pathway in the glioblastoma cell line [30]. In our research, the phosphorylation of STAT3 was reduced by Src and Pyk2 inhibitors and did not respond to PI3K inhibitor, suggesting that CCR7 activates STAT3 via Src and pyk2 in SCCHN, and PI3K/Akt does not participate in this STAT3 activation.

To address how this CCR7/JAK2/STAT3 signal pathway mediates SCCHN metastasis, we investigated two epithelial mesenchymal transition- (EMT-) related molecules. E-cadherin is a transmembrane glycoprotein associated with the cytoskeleton via cytoplasmic proteins. The disruption of E-cadherin-mediated adhesion is considered a key step in the progression toward the malignant phase of carcinoma, which has been observed to be induced by many signal pathways [31]. In colorectal cancer, STAT3 induced cell invasion and downregulation of E-cadherin, thus promoting tumor EMT [32]. Our study had demonstrated that CCR7/JAK2/STAT3 regulating SCCHN metastasis can also occur via E-cadherin. Vimentin is the major intermediate filament (IF) protein of mesenchymal cells, and it is associated with EMT. In breast cancer cells, the pharmacological inhibition of STAT3 activation using a small molecule inhibitor, Stattic, potentiated HNK-mediated inhibition of vimentin [33]. In our study, although AG490 can inhibit STAT3 phosphorylation, it cannot disrupt the CCL19-induced expression of vimentin, suggesting that vimentin is not downstream of CCR7/JAK2/STAT3.

In gastric cancer, pSTAT3 expression was significantly higher than in adjacent nontumor tissue, and there was a significant association between pSTAT3 expression and lymph node metastasis [34]. In our immunohistochemical staining study, pSTAT3 was also highly expressed in SCCHN tumor tissue and was correlated with tumor cervical lymph node metastasis and clinical stage. Furthermore, we also analyzed the relationship between CCR7 and p-STAT3 expression and found that there is a positive correlation between them. These results can be helpful for demonstrating that CCR7 activates JAK2/STAT3 in SCCHN and that this pathway played a critical role in promoting tumor metastasis, which we demonstrated in vitro.

## 5. Conclusion

Taken together, our study supports a novel hypothesis that CCR7 can activate JAK2/STAT3 signal pathways in SCCHN that are dependent on Src and Pyk2. This CCR7/JAK2/STAT3 signal pathway regulates tumor metastasis by E-cadherin-mediated tumor EMT. In this regard, our results will improve the study of CCR7 in SCCHN and provide an important experimental basis for developing therapeutics to treat SCCHN in the future.

## Figures and Tables

**Table 1:** Correlations between CCR7, STAT3 phosphorylation expression and clinicopathological factors of SCCHN.

Clinicopathological characteristics	Number of cases	CCR7		Statistical analysis	STAT3 phosphorylation		Statistical analysis
		+~+++	−		+~+++	−	
Age							
≥60	40	25	15	*χ* ^2^ = 0.032	25	15	*χ* ^2^ = 0.422
<60	38	23	15		21	17	
Gender							
Male	50	32	18	*χ* ^2^ = 0.357	30	20	*χ* ^2^ = 0.061
Female	28	16	12		16	12	
Tumor size							
T1, T2	65	37	28	*χ* ^2^ = 3.510	36	29	*χ* ^2^ = 2.077
T3, T4	13	11	2		10	3	
Clinical stage							
I, II	37	15	22	*χ* ^2^ = 13.113∗	14	23	*χ* ^2^ = 12.998∗
III, IV	41	33	8		32	9	
Nodal metastasis							
Yes	37	29	8	*χ* ^2^ = 8.434∗	29	8	*χ* ^2^ = 10.954∗
No	41	19	22		17	24	

^*^ *P* < 0.05 (the internal difference of CCR7 or STAT3 phosphorylation expression within clinicopathological characteristics).

**Table 2:** The correlations between CCR7 and STAT3 phosphorylation expression in SCCHN primary tumor (*r* = 0.327, *P* = 0.003).

		STAT3 phosphorylation	
		+~+++	−
CCR7	+~+++	33	15
	−	13	17
